# Recent strategies for enhancing the therapeutic efficacy of stem cells in wound healing

**DOI:** 10.1186/s13287-021-02657-3

**Published:** 2021-11-25

**Authors:** Yongqing Zhao, Min Wang, Feng Liang, Jiannan Li

**Affiliations:** Department of General Surgery, The Second Hospital of Jilin University, No. 218 Ziqiang Street, Changchun, 130041 Jilin China

**Keywords:** Wound healing, Embryonic stem cells, Adult stem cells, Induced pluripotent stem cells, Mesenchymal stem cells

## Abstract

Skin wound healing is a multi-stage process that depends on the coordination of multiple cells and mediators. Chronic or non-healing wounds resulting from the dysregulation of this process represent a challenge for the healthcare system. For skin wound management, there are various approaches to tissue recovery. For decades, stem cell therapy has made outstanding achievements in wound regeneration. Three major types of stem cells, including embryonic stem cells, adult stem cells, and induced pluripotent stem cells, have been explored intensely. Mostly, mesenchymal stem cells are thought to be an extensive cell type for tissue repair. However, the limited cell efficacy and the underutilized therapeutic potential remain to be addressed. Exploring novel and advanced treatments to enhance stem cell efficacy is an urgent need. Diverse strategies are applied to maintain cell survival and increase cell functionality. In this study, we outline current approaches aiming to improve the beneficial outcomes of cell therapy to better grasp clinical cell transformation.

## Introduction

As a main organ of the human body, the skin is the first link between the human body and the outside world [1]. The most important function of the skin is to prevent some mechanical, physical, and chemical damage and block the invasion of bacteria. The integrity of skin is the prerequisite for maintaining its function. Once the body is severely damaged or due to internal abnormalities such as diabetes and vascular insufficiency, normal skin physical structure or functional stability is disrupted. Thus, wounds are formed, even chronic or non-healing wounds.

**Fig. 1:**
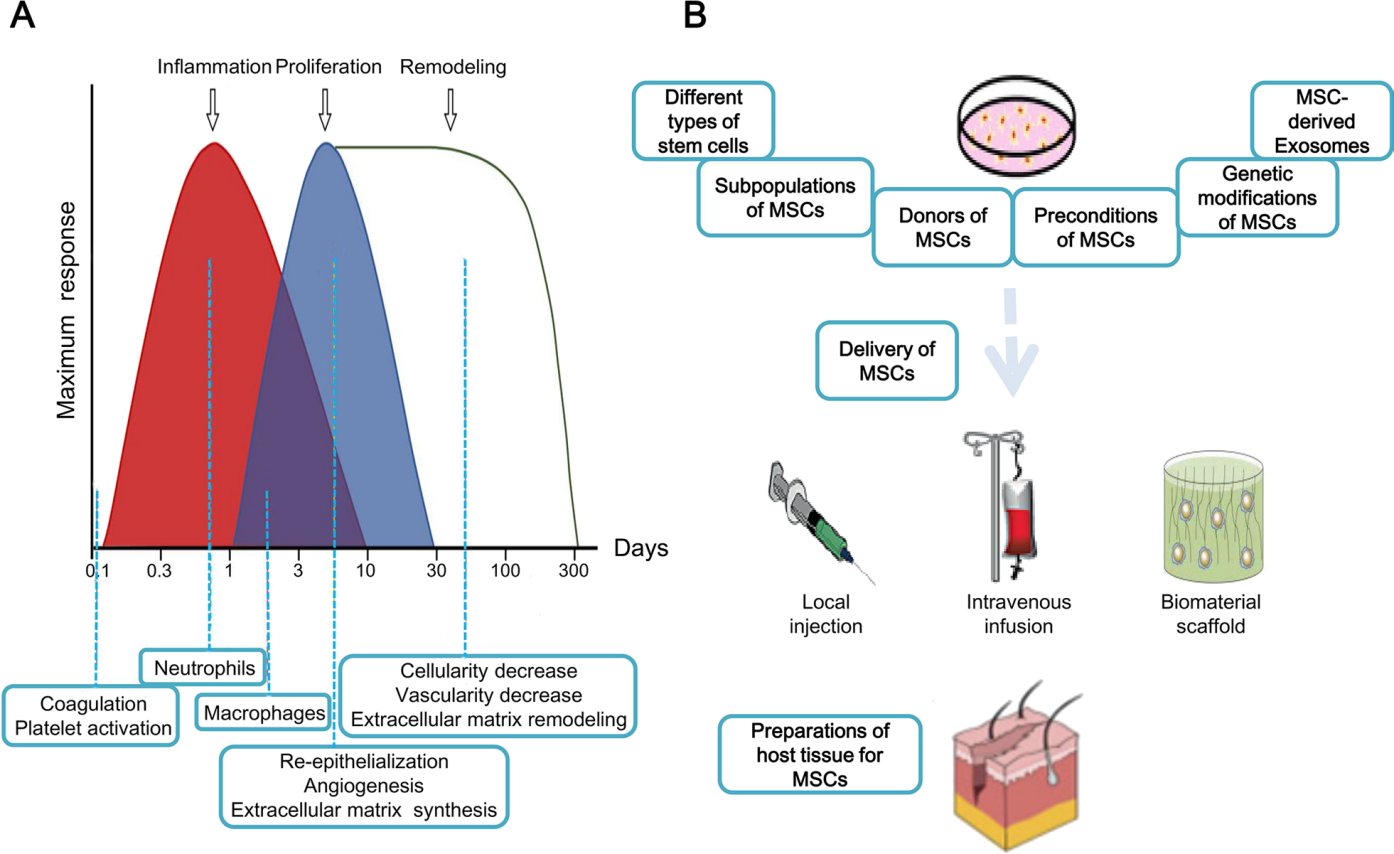
Wound healing process and different ways for stem cells to enhance the treatment efficacy of wound healing. **A** Timeline of skin wound healing [2]; **B** different ways of stem cells to enhance the treatment efficacy of wound healing [3]. Reproduced from the article by authors Casado-Díaz et al. [2], copyright 2020, Casado-Díaz et al. Reproduced from the article by authors Baldari et al. [3], copyright 2017, Baldari et al.

Normal skin wound healing is a dynamic system that relies on multiple cells and mediators communicating in a rather complicated time series after injury. The main phases of wound healing include inflammation, proliferation, and remodeling (Fig. 1A) [2]. When an injury occurs, the platelets are triggered to form a clot to close the wound and limit the bleeding. Meanwhile, the leukocytes are recruited, and the inflammatory phase plays a role in fighting bacterial infections. As inflammation diminishes, epithelial cells begin to proliferate and keratinocytes migrate to promote epithelialization. In the final remodeling phase, the extracellular matrix (ECM) is constantly reconstructed and the proportion of various kinds of collagen changes to strengthen the skin resilience.

In fact, unadvanced wound healing can occur in any phase of skin recovery due to abnormal factors. Confined to a prolonged inflammatory stage, chronic wound is exposed to persistent bacterial infections and excessive proinflammatory cytokine stimulation, which requires constant treatment. Chronic wound, such as pressure sores, diabetic ulcers, and arteriovenous ulcers, not only lowers the living quality of patients but also imposes a huge economic burden on society. In addition, the poor appearance of wounds and the inconveniency of movements both bother patients.

Therefore, various therapies have been developed to manage chronic wounds, of which traditional therapies are favored for debriding necrotic tissue, applying wound dressings, using antibiotics, and performing skin graft if necessary. As for emerging therapies, some biophysical modalities, such as electrical stimulation and shock wave therapy, are used to faster wound regeneration. Besides, engineered skin substitutes are popular in the tissue regeneration. They can be seeded with keratinocytes and/or fibroblasts to replace the recipients’ defective skin. Recently, stem cell therapy has received increasing attention in wound healing due to its excellent abilities in self-renewal, differentiation, and immunomodulation.

Although significant progress has been made on stem cell treatment for cutaneous wound healing, the potentials of stem cells remain to be unleashed. The transplanted stem cells have a short duration of existence and a low survival rate at the wound site. When the cell loses its original supportive environment, apoptotic signaling is activated, leading to the death of cells. Besides, the mechanical stress exerted on the cells during delivery, and the harsh conditions of host after translation both affect the cell viability. Therefore, one of the aims to optimize cell therapy is to increase cell survival. Additionally, promoting cell functionality is another goal. In this study, we summarize current optimizing strategies to enhance the wound healing efficacy of stem cells (Fig. 1B) [3].

## Strategies to promote favorable effects of cell therapy

### Different types of stem cells

**Fig. 2:**
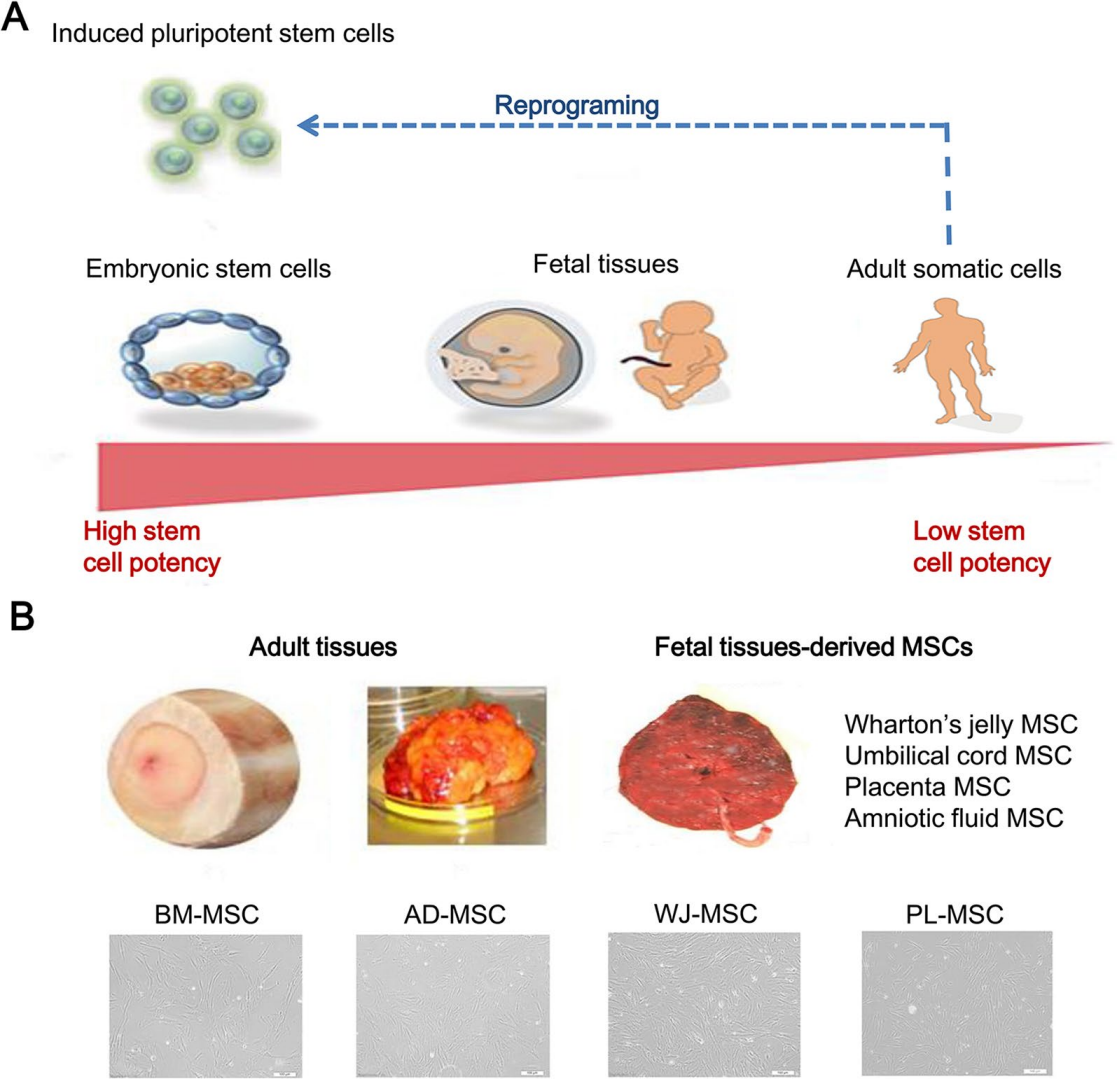
Differentiation potential of different stem cells and the sources of MSCs. **A** Differentiation potential of different stem cells types [4]; **B** different sources of MSCs and their cell morphologies [6]. BM-MSC: bone marrow-derived mesenchymal stem cells; AD-MSC: adipose tissue-derived mesenchymal stem cells; WJ-MSC: Wharton’s jelly-derived mesenchymal stem cells; PL-MSC: placenta-derived mesenchymal stem cells. Reproduced from the article by authors Duscher et al. [4], copyright 2015, Karger Publishers, Basel, Switzerland. Reproduced from the article by authors Li et al. [6], copyright 2014, Li et al.

A relatively effective stem cell source is the starting point for optimal outcomes because multiple types of stem cells have different wound healing effects. Besides, advantages and limitations both exist in each type of stem cells. Stem cells are classified into embryonic stem cells (ESCs), adult stem cells (ASCs) and induced pluripotent stem cells (iPSCs). These stem cells show different differentiation potential, among which ESC and iPSCs have higher differentiation potential compared to ASCs (Fig. 2A) [4]. ASCs include multiple types of stem cells, such as mesenchymal stem cells (MSCs), hematopoietic stem cells, and umbilical cord stem cells. A brief comparison of the characteristics of ESC, iPSCs, and ASCs (mainly MSCs) is presented in Table 1. Among ASCs, MSCs have been applied more widely and successfully for the treatment of many kinds of diseases, including wound healing. As a result, we mainly highlight the comparison of MSCs from different sources in the treatment of wound repair.

**Table 1:** The main characteristics of ESC, iPSCs, and ASCs are compared from five aspects

	Source	Differentiation potential	Advantages	Disadvantages	Prospects
Embryonic stem cells (ESCs)	Blastocyst	Pluripotent (differentiation ability to any cell type of three germ layers)	1. Unlimited proliferation capacity 2. Strong differentiation potential	1. Immunogenicity 2. Tumorigenicity 3. Ethical dilemmas and regulatory issues 4. Invasive harvesting method	Potential of forming an entire organism
Induced pluripotent stem cells (iPSCs)	Adult somatic cells	Pluripotent (differentiation ability to any adult cell type)	1. Rich and easily accessible source 2. High differentiation capacity 3. Negligible immune responses (syngeneic iPSCs) 4. No ethical controversy	1. Possibilities of tumor formation 2. Possible genetic and epigenetic abnormalities during reprogramming 3. Immunogenicity (allogeneic iPSCs)	Possible genetic defects that can be modified during reprogramming
Adult stem cells (ASCs), mainly mesenchymal stem cells (MSCs)	Differentiated tissues and organs	Multipotent (directed differentiation of multiple cell types)	1. Strong self-renewal ability 2. Limited immunoreactivity and certain immunoregulatory property 3. Controllable security and low tumorigenicity 4. Readily available, non-invasive, and less polluted acquisition of fetal tissue-derived MSCs	1. Invasive obtainment of BM-MSCs and AT-MSCs 2. The most suitable cell source has not been determined 3. Limited cell efficacy and underutilized therapeutic potential	1. The clinical application of MSCs, especially BM-MSCs, is more successful and extensive 2. Fetal tissue-derived MSCs have displayed promising outcomes 3. The application of exosomes presents a cell-free therapy

The minimum standard for MSCs has been established by the International Society for Cellular Therapy (ISCT) with respect to cell culture characteristics, differentiation potential, and surface molecular expression [5]. MSCs can be present in almost any human tissue, in which bone marrow-derived (BM), adipose tissue-derived (AD), and fetal tissues-derived (Wharton’s jelly (WJ), umbilical cord, placenta, and amniotic fluid) MSCs draw more attention (Fig. 2B) [6]. MSCs from these adult or fetal tissues display a fibroblast-like morphology (Fig. 2B) [6]. Their differentiation potentials are considered as a mechanism in regenerative medicine. However, it is accepted that the bioactive molecules secreted by paracrine signaling of MSCs play a pivotal role [7]. The main beneficial effects of bioactive molecules responsible for the regeneration of tissue are immunomodulation, angiogenesis, and others. In the inflammatory phase of injury, MSCs participate in regulating immune response by influencing the function of various immune cells. The immunomodulatory capacities are not exactly the same in different types of MSCs. For example, Li et al. compared the immune properties of MSCs from four sources (BM, AD, WJ, and placenta), demonstrated that WJ-MSCs could be applied in requirement of immunosuppressive action as the most suitable cell type with the strongest T cell inhibition and the weakest immune-related gene expression [6]. Apart from immunomodulation, there is heterogeneity in proangiogenic features of MSCs. A study revealed that BM-MSCs and placental MSCs gave priority to promoting angiogenesis, because more angiogenic genes expressed and more growth factors were produced compared to those of umbilical cord (UC)-MSCs and AD-MSCs [8]. However, Han et al. regarded that placenta chorionic villi-derived MSCs were more efficient in angiogenesis and immunomodulation than BM-, UC-, and AD-MSCs [9].

The controversies in this field need more investigation. As a result, no single type of stem cell has been displayed to be optimal for wound regeneration. The type of MSCs required depends on the specific situation due to different cell sources. Nonetheless, fetal tissue-derived MSCs have certain advantages in improved capacities on proliferation, immunomodulation, angiogenesis, and scarless wound healing [10], which are attractive candidates in tissue regeneration.

### Subpopulations of MSCs

Interest has increased hugely in the heterogeneity of stem cell populations. Cell populations of the same type from different donors and tissue sources differ in phenotypes and functions [11]. Scientists refer to heterogeneous cell populations as subpopulations. Even from the same tissue of the same individual, cell populations have different surface marker expression and exhibit distinct features [11]. Identifying subpopulations we need in these cell populations is a promising direction to enhance the efficacy of stem cells. Therefore, single-cell RNA sequencing, as a novel and powerful technology, has been applied to characterize the heterogeneity of cell populations at the single-cell level and can efficiently analyze the gene expression profile of various heterogeneous populations in large quantities with no difference [12]. In this way, the subpopulations with common gene expression can be identified and selected.

**Fig. 3:**
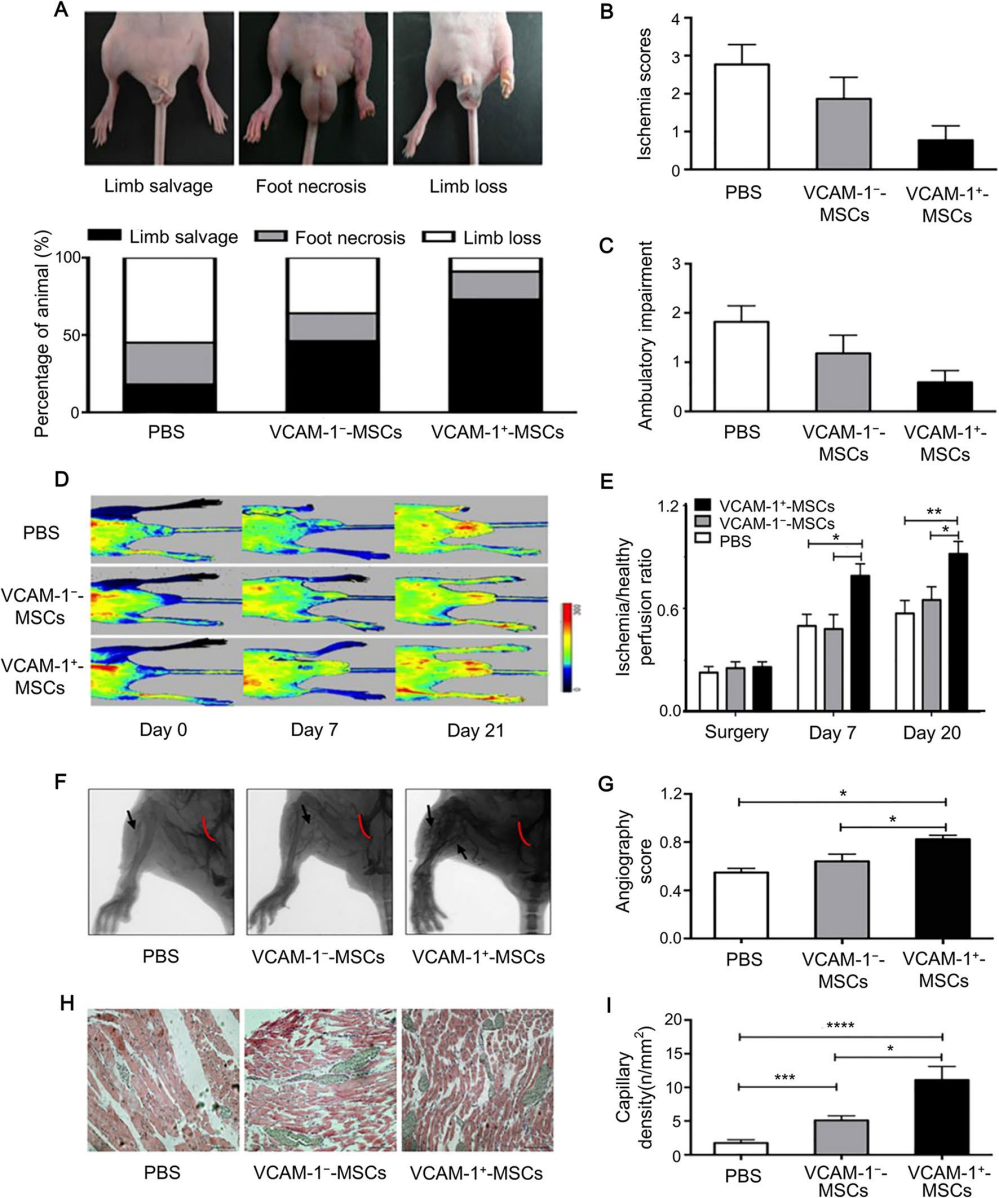
Improved blood perfusion and increased formation of collateral vessels in ischemic site after the transplantation of VCAM-1 + CV-MSCs [15]. **A** Different proportions of limb salvage, foot necrosis, and limb loss in VCAM-1 + / − CV-MSCs and PBS groups; **B** ischemia scores and **C** ambulatory impairment scores are used to assess the ischemia injury and the function of ischemic limbs; **D** different conditions of blood perfusion (red represents increased perfusion) in three groups; **E** the blood flow restoration of three groups is measured by blood perfusion ratio; **F** the formation of vessels in three groups is evaluated by angiography and **G** their angiography scores are shown; **H** H & E staining is used to further confirm; **I** vessel density of ischemia limbs. CV: chorionic villi; PBS: phosphate-buffered saline; VCAM-1: vascular cell adhesion molecule 1. Reproduced from the article by authors Du et al. [15], copyright 2016, Du et al.

Utilizing single-cell RNA sequencing, Sun et al. investigated different subpopulations of WJ-MSCs and distinguished six clusters (C0–C5) with distinct features [13]. Notably, CD142 and other multiple genes of skin repair in the C3 cluster are expressed, suggesting a recovery potential for wound healing. In this study, further evidence has demonstrated that the healing potency of CD142^+^ WJ-MSCs is stronger than that of CD142^−^ WJ-MSCs. CD142^+^ WJ-MSCs present an opportunity to improve the cell efficacy of skin injury treatment. Besides, Rennert et al. demonstrated that a cell subpopulation expressing DPP4 and CD55 could enhance cell survival and proliferation [14]. To further assess its outcome, the treatment with enriched subpopulation was performed in the diabetic wounds of mice, showing accelerated healing time relative to that with the depleted subpopulation. Thus, this subpopulation could be selected as an efficient and beneficial factor for cell retention. Furthermore, in terms of angiogenesis and immunomodulation, Han et al. reported that a subpopulation of VCAM-1^+^ (CD106) MSCs, originating from the placenta chorionic villi exhibited potential advantages relative to that of VCAM-1^−^ MSCs [9]. The enhanced immunomodulatory and proangiogenic behaviors of VCAM-1^+^ MSCs are essential for therapeutic applications. These superior features in certain subpopulations enable encouraging outcomes in the treatment of tissue regeneration. For instance, Du et al. found a significant functional improvement in the mice’ ischemic limb after the transplantation of VCAM-1^+^ MSCs, assessed by several different indexes (Fig. 3) [15]. The VCAM-1^+^-MSCs-treated mice had less ischemia damage and ambulatory impairment compared to control groups. Besides, the authors identified that VCAM-1^+^ MSCs exerted more therapeutic effects on ischemia site, which were evaluated by the ischemia restoration and the formation of collateral vessels. Selecting the subpopulation with superior pro-angiogenic effects for wound regeneration by using VCAM-1 as a biomarker is valid. Therefore, identifying and enriching the subpopulation with required functional features by biomarker recognition increases the efficacy of stem cells in wound treatments.

### Donors of MSCs

The properties of MSCs derived from various donors are varied as well. According to the donor source, there are two cell types classified as syngeneic and allogeneic MSCs, which have been applied successfully in wound regeneration.

Syngeneic MSCs are obtained from the donor who is genetically identical to the recipient; that is, cells are from the same individual. The threat of an allogeneic immune response, therefore, is not considered. However, their isolation, in terms of cell quality and quantity, can be affected by the health conditions and age factors of donors. Wang et al. observed a physical dysfunction in mice treated with the transplantation of AD-MSCs from aged donors rather than young donors [16]. Aging or impaired MSCs are limited to exert their functions, and more importantly, if, in an emergency, MSCs from patients themselves are not immediately available because it takes a long time to obtain qualified cell products. Under these circumstances, the application of allogeneic MSCs can meet urgent needs.

Allogeneic MSCs are collected from other donors prior to their application and possess an “off the shelf” therapeutic feature. However, the safety issues around allogeneic MSCs have been one of the constant concerns. Accumulated evidence revealed that the transplanted allogeneic MSCs could induce variable immune responses of the host. In an equine model, Joswig et al. compared immune responses induced by injecting syngeneic and allogeneic BM-MSCs into animal joints, respectively, and found that the joint of equine produced a significant adverse reaction after repeated intra-articular injection of allogeneic BM-MSCs [17]. The results of pre-clinical animal models deserve more attention to prevent the same adverse reaction in humans.

**Fig. 4:**
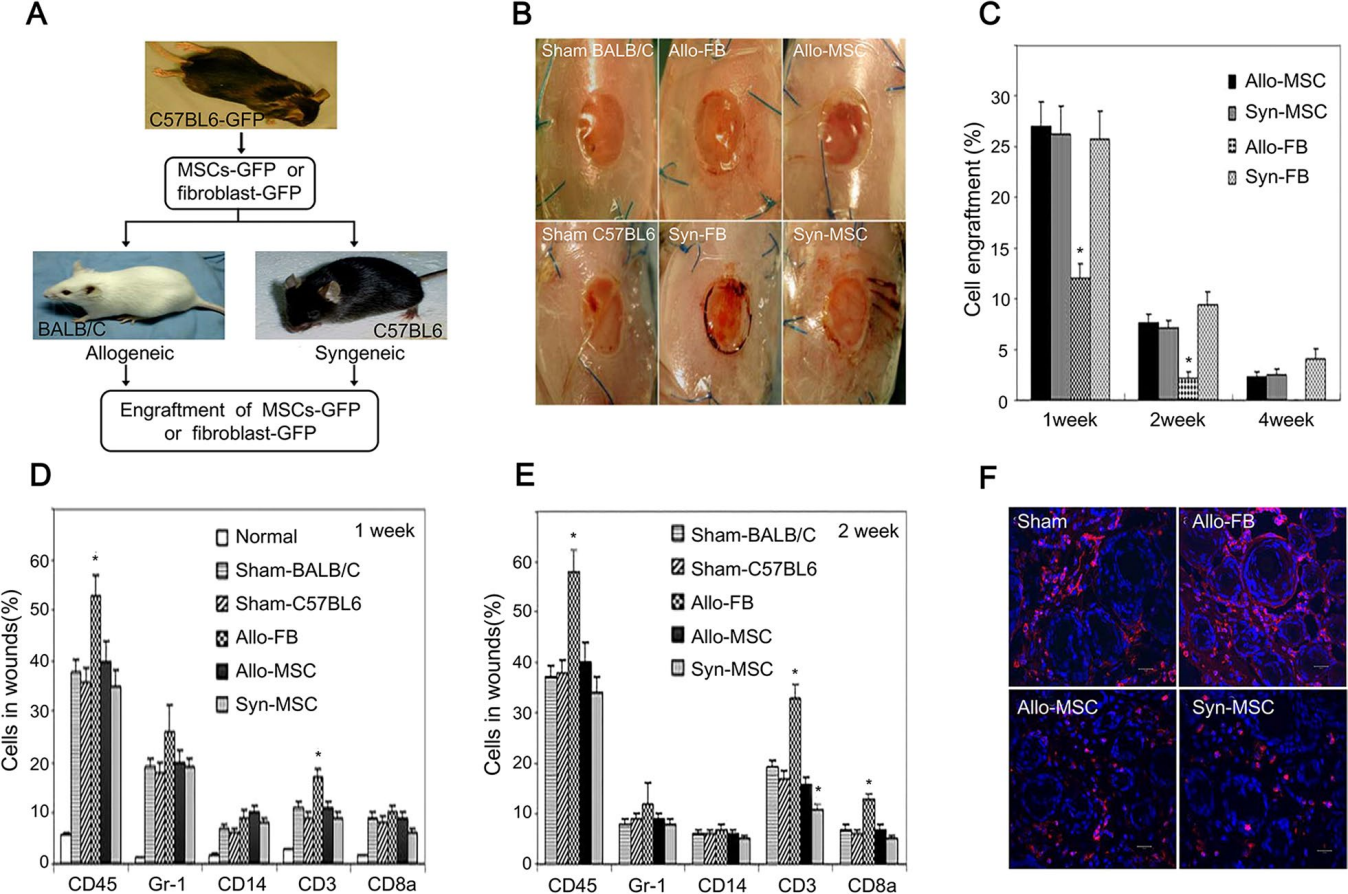
Comparison of the effects of allogeneic and syngeneic MSCs in wound regeneration [18]. **A** Experimental scheme and the method of obtaining allogeneic or syngeneic GFP + MSCs and GFP + fibroblasts; **B** the effects of transplanted MSCs, fibroblasts or control medium (sham) derived from C57BL/6 mice on excisional wounds in BALB/C or C57BL/6 mice; **C** the engraftment of MSCs or fibroblasts in wounds; **D**, **E** the proportions of leukocytes or their subsets in the normal skin or wounds at 1 week or 2 weeks; **F** the images of CD3 + T cells (red) in wounds at 14 days. Allo-FB: allogeneic fibroblast; Syn-FB: syngeneic fibroblast; Allo-MSC: allogeneic mesenchymal stem cells; Syn-MSC: syngeneic mesenchymal stem cells. Reproduced from the article by authors Chen et al. [18], copyright 2009, Chen et al.

However, it was also reported that allogeneic MSCs possessed negligible immunogenicity and comparable efficacy with syngeneic MSCs. Chen et al. found similar amounts of implanted syngeneic and allogeneic BM-MSCs in excisional wounds of mice, indicating that the host immune response did not affect the survival of allogeneic cells [18]. Allogeneic and syngeneic MSCs were equally efficient in promoting wound closure (Fig. 4) [18]. Besides, they compared the reactions of allogeneic-MSCs and allogeneic-fibroblasts in wounds. Leukocytes were increased in allogeneic-fibroblasts-treated wounds. The authors concluded that the reduced cell engraftment was due to the immune response induced by allogeneic fibroblasts rather than allogeneic BM-MSCs. Chang et al. assessed the healing efficacy of syngeneic and allogeneic AD-MSCs on the burn wounds of rats and observed that tissue repair in the allogeneic and control groups showed no significant differences, while it was faster in the syngeneic AD-MSCs group [19]. These different results were probably due to the selection of stem cell types and experimental models.

Consequently, allogeneic MSCs can treat skin wounds if the effects of immune rejection are kept under control. The convenience and availability of allogeneic cell transplantation make the application in wound regeneration more practical. As for syngeneic MSCs, a feasible consideration is to establish and expand the cryobank of stem cells in advance, such as the cryopreservation of fetal tissue-derived cells, making it possible for future use of syngeneic MSCs in any situation.

### Precondition methods

**Fig. 5:**
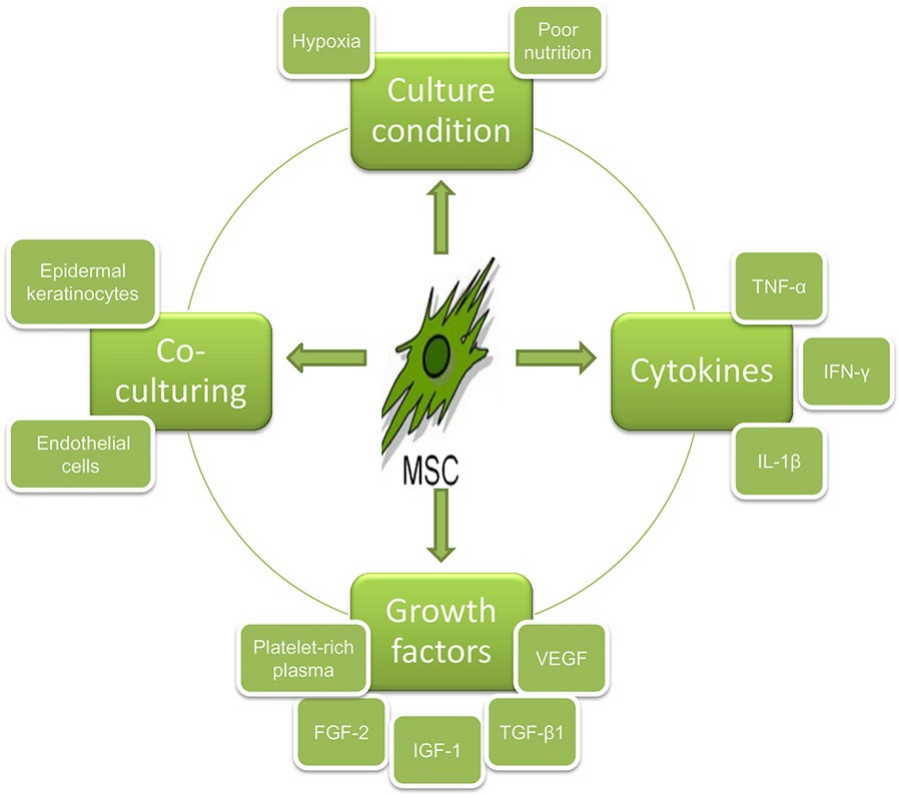
Various preconditioning strategies for MSCs by changing the culture conditions and providing additional pretreatments [20]. TNF-α: tumor necrosis factor-α; IL-1β: interleukin-1β; IFN-γ: interferon-γ; PRP: platelet-rich plasma; FGF-2: fibroblast growth factor-2; IGF-1: insulin-like growth factor 1; TGF-β1: transforming growth factor-β1; VEGF: vascular endothelial growth factor. Reproduced from the article by authors Hu et al. [20], copyright 2018, Hu et al.

Preconditioning strategies have been investigated to maintain cell survival and improve cell efficacy in various studies. Culturing MSCs in different environments and patterns, and pretreating MSCs with different cytokines, growth factors, or some cells in advance improves the therapeutic efficacy of MSCs in tissue regeneration (Fig. 5) [20].

**Fig. 6:**
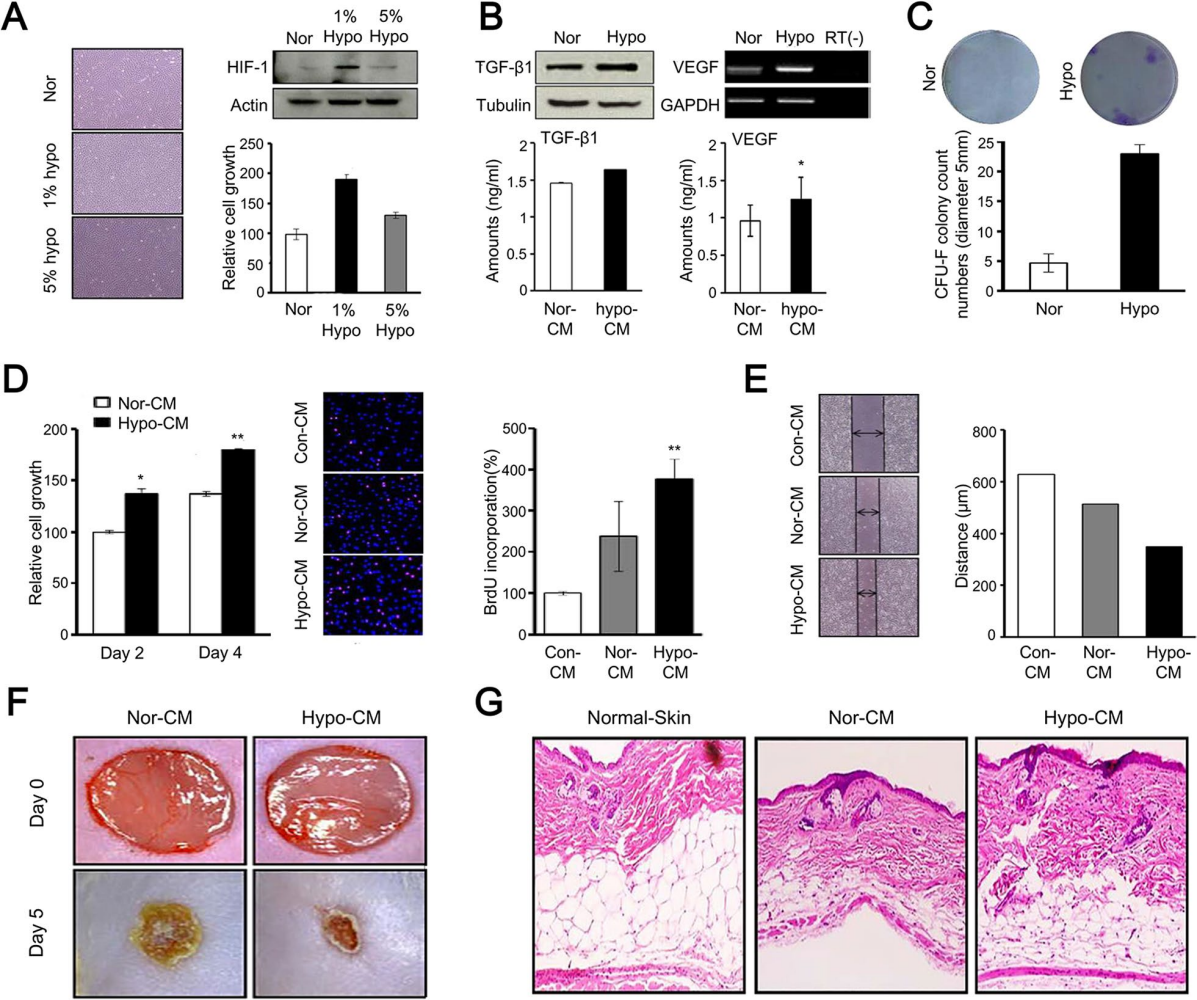
The effects of hypoxia on MSCs and the effects of their hypoxic conditioned media on fibroblasts and wound closure [22]. **A** The growth of MSCs and the expression of HIF1-α protein under normoxic or hypoxic conditions (1% or 5% O2); **B** the expression levels of TGF-β1 and VEGF were confirmed in MSCs and their conditioned media; **C** the clonogenic capacity of MSCs was detected by CFU-F assay; **D** BrdU assay was performed to determine the growth of fibroblasts in different conditioned media; **E** scratch-wound-closure assays were performed to confirm the migration of fibroblasts at the wound site; arrows show the distance of wound; **F** the images of wounds size; **G** histomorphometric analysis of wound closure. HIF-1α: inducible transcription factor 1α; nor-CM: normoxic conditioned media; hypo-CM: hypoxic conditioned media; con-CM: conditioned media; CFU-F: colony-forming unit fibroblast; TGF-β: transforming growth factor-β; VEGF: vascular endothelial growth factor. Reproduced from the article by authors Jun et al. [22], copyright 2014, Jun et al.

Culture condition is important for cell growth and development. The environment of the wound site lacks oxygen and nutrients, thus not as suitable as the original living or culturing environment of MSCs. It is possible to change the cell culture condition before cell implantation to regulate cell metabolic activities. By culturing cells in a low energy requirement state, the cells can adapt to the harsh environment of the wound in advance, thus providing defensive protection for cell activities. A study showed that hypoxic preconditioning (1% O2) of BM-MSCs could improve cell viability after cell transplantation in mice [21]. A hypoxic environment maintained these cells in a low glucose consumption state so that these cells could survive longer than untreated BM-MSCs. Jun et al. revealed that hypoxia preconditioned (1% O2) amniotic fluid (AF)-derived MSCs possessed improved cell proliferation and enhanced secretion of paracrine factors relative to AF-MSCs under normoxic conditions [22]. Their hypoxic conditioned media were demonstrated to promote the proliferation and migration of fibroblasts and accelerate skin wound healing (Fig. 6) [22]. Apart from hypoxia preconditioning, subjecting cells to low nutrient supply in advance could also affect cell vitality. By depriving the support of plasma, Moya et al. induced BM-MSCs into a quiescent condition while preserving the multipotential capabilities [23]. These cells were implanted into the ischemic tissue of mice, which exhibited improved cell viability in vivo*.* Therefore, mimicking the condition of the wound environment by providing low supports of oxygen and nutrients for cells before implantation is beneficial for cell survival. The efficacy of cells can be significantly affected by the culturing condition.

Culturing cells in three-dimensional (3D) aggregation can also preserve cell survival and properties. The 3D aggregate of MSCs is a spheroid formed by 500–10,000 cells, which depends on the mutual recognition of cadherin on the cell surface [24]. The aggregation of MSCs can maintain the intercellular interaction and cell-ECM connection in cell culture, thereby preventing cells from apoptosis. Besides, more ECM proteins and angiogenic factors can be produced by 3D aggregates of MSCs in wound regeneration. For instance, compared to cell suspensions, 3D cell aggregates show elevated ECM secretions and enhanced wound closure in diabetic wounds of mice [25]. The activities of MSCs can be significantly affected by the way of cell formation. Better neovascularization of ischemic tissue can be achieved by 3D cell aggregates through promoting cell survival and angiogenesis [26]. Therefore, culturing MSCs by 3D aggregation is an effective strategy to enhance cell therapeutic outcomes.

In addition to changing the conditions and patterns in cell culture, the pretreatments of MSCs are also applied to improve therapeutic efficacy. At the injury site, transplanted cells are exposed to an inflammatory environment, and the enhancement of cellular immunomodulatory function should also be emphasized. Preconditioning MSCs with proinflammatory cytokines, such as tumor necrosis factor-α (TNF-α), interleukin-1β (IL-1β), and interferon-γ (IFN-γ), augments immunomodulatory properties. IFN-γ-preconditioned MSCs could inhibit T-cell and T-cell effector and enhance wound repair in mice [27]. IL-1β is an inflammatory mediator, and IL-1β-treated MSCs could upregulate gene expression related to immunomodulation [28]. Moreover, a recent study assessed the immunotherapeutic function of MSCs by combinatory preconditioning with hypoxia and proinflammatory cytokines (TNF-α, IL-1β, IFN-γ), which showed a robust anti-inflammatory effect [29]. Nonetheless, this combinatory precondition appears not to be the most suitable treatment because of the impairment on cell differentiation and self-renewal. Therefore, the rational use of different inflammatory cytokines needs more researches.

Growth factors are also used for the precondition MSCs, and platelet-rich plasma (PRP), containing multiple growth factors, is more explored to provide trophic support to cells. Hersant et al. reported that the treatment of combining MSCs with PRP could promote angiogenic, survival, and proliferative potential of MSCs, contributing to increased wound healing rate and skin elasticity in a mouse wound model [30]. Different growth factors have unique functions as well as common effects. Fibroblast growth factor-2 (FGF-2) can promote the differentiation and proliferation of AD-MSCs [31]. Insulin-like growth factor 1 (IGF-1) has been demonstrated to improve implanted cell viability and increase cell resistance to apoptosis [32]. Transforming growth factor-β1 (TGF-β1) has a promoting effect on the proliferation of human UC-MSCs and the expression of ECM genes [33]. Vascular endothelial growth factor (VEGF) is beneficial for the vascularization of the engineered dermis [34]. The effects of each growth factor are interactive, and it is necessary to explore the mixture of different types of growth factors to maximize their functions.

Co-culturing MSCs with other cells is also proved to increase cell efficacy. Seo et al. assessed the effects of AD-MSCs co-cultured with human epidermal keratinocytes and found a higher proliferation and epithelial differentiation of AD-MSCs relative to monoculture AD-MSCs [35]. In a 3D scaffold, Freiman et al. co-cultured AD-MSCs with microvascular endothelial cells to investigate their integrated angiogenic potential, which showed promoted vascular network formation [36]. The addition of other cells to the culture environment can enhance the contacts of cells and increase the therapeutic properties of MSCs.

### Genetic modifications

**Fig. 7:**
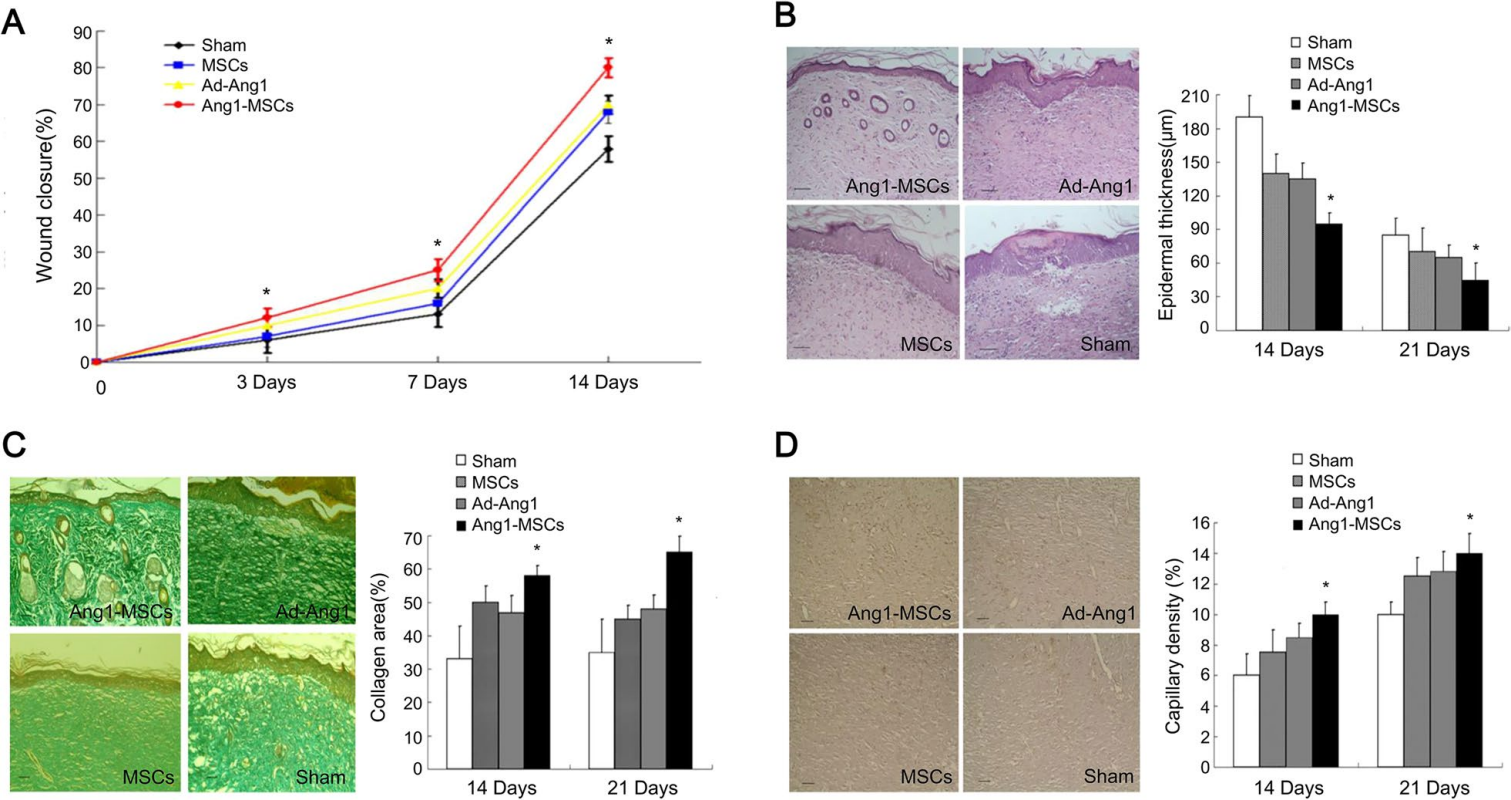
The effects of angiopoietin-1 gene-modified MSCs (Ang1-MSCs) on wound healing [39]. Excisional wounds of rats received treatment with Ang1-MSCs, MSCs, recombinant adenovirus encoding angiopoietin-1 (Ad-Ang1), and vehicle medium (sham). **A** Wound closure of different treatments; **B** histological images of the wounds with different treatments and the comparison of epidermal thickness; **C** Masson’s trichrome-stained images of the wounds with different treatments and the comparison of collagen deposition; **D** the images of CD31 (a blood vessel endothelial cell marker) staining (yellow) in wounds with different treatments and the comparison of capillary density. Ang1: angiopoietin-1; Ad-Ang1: recombinant adenovirus encoding angiopoietin-1. Reproduced from the article by authors Li et al. [39], copyright 2013, Li et al.

Genetic modification is to treat skin wounds by inserting specific genes into host cells. Nowadays, MSCs have become the genetic target to be modified to increase their retention and reinforce their efficacy in tissue regeneration. Song et al. modified and induced AD-MSCs to express v-myc gene, which endowed cells with high growth potential and increased their maintenance time [37]. In these v-myc AD-MSCs, protein kinase B (Akt) gene was induced to be expressed in determining their paracrine effects in wound repair. Researchers found that v-myc-Akt AD-MSCs improved cell survival and increased secretion of growth factors, accelerating wound closure. Stromal-derived factor-1 (SDF-1) and C-X-C chemokine receptor 4 (CXCR4), in a signaling pathway, play a critical role in cell migration and homing. By overexpressing CXCR4 in BM-MSCs of mice, Yang et al. found that the time of wound regeneration was significantly reduced owing to the increased cell recruitment in wound tissue and identified that the behavior of cell migration depended on the expression of SDF-1 [38]. Moreover, the angiogenic property of MSCs can be enhanced by modifying related genes. A study showed that angiogenesis and skin regeneration was significantly promoted by angiopoietin-1 gene-modified BM-MSCs (Ang1-MSCs) [39]. The wound treated with Ang1-MSCs, had thinner epidermal thickness, higher capillary density, and a more arranged collagen network (Fig. 7) [39]. Modifying Ang1 gene of MSCs increased the efficiency of wound repair.

Collectively, engineering MSCs to deliver genes of interest represents a promising optimized strategy for cell-based therapy. Genes beneficial for cell survival, cell migration, and tissue angiogenesis need more exploration.

In addition to modifying some target genes, manipulating microRNA (miRNA) is also an approach to regulate gene expression in many cellular processes of tissue repair, thereby controlling the functions of related genes. Miscianinov et al. revealed that miRNA-148b was associated with endothelial cell homeostasis via TGF-β pathway, and applying mimics of miRNA-148b could drive angiogenesis and stimulate wound closure in a mouse model [40]. Xu et al. reported that miRNA-146a was a critical factor in inflammatory responses and the treatment of MSCs with reduced miRNA-146a probably resulted in chronic inflammation in a diabetic wound [41]. These pieces of evidence indicate that cell efficacy can be improved by manipulating some miRNAs.

## MSC-derived exosomes

MSC-derived exosomes can translate cell-based therapy into cell-free therapy. The effects of translated MSCs on tissue regeneration are determined by paracrine abilities rather than differentiation. Mounting studies have confirmed that conditioned medium consisting of various MSCs secretomes possesses similar therapeutic effects with MSCs in tissue regeneration [42]. Especially, the membrane structures of the cytoplasm and the multivesicular bodies (MVBs) can fuse to secret exosomes, a kind of secretary extracellular vesicles (EVs). MVBs are formed by invagination of the plasma membrane. Exosomes have a delivery capacity to transfer functional cargo molecules that contain a variety of complicated RNAs and proteins, exerting essential effects on the communication between cells and the mediation of paracrine. The beneficial effects of exosomes have garnered significant attention and have been confirmed for their effective applications in enhancing tissue repair.

**Fig. 8:**
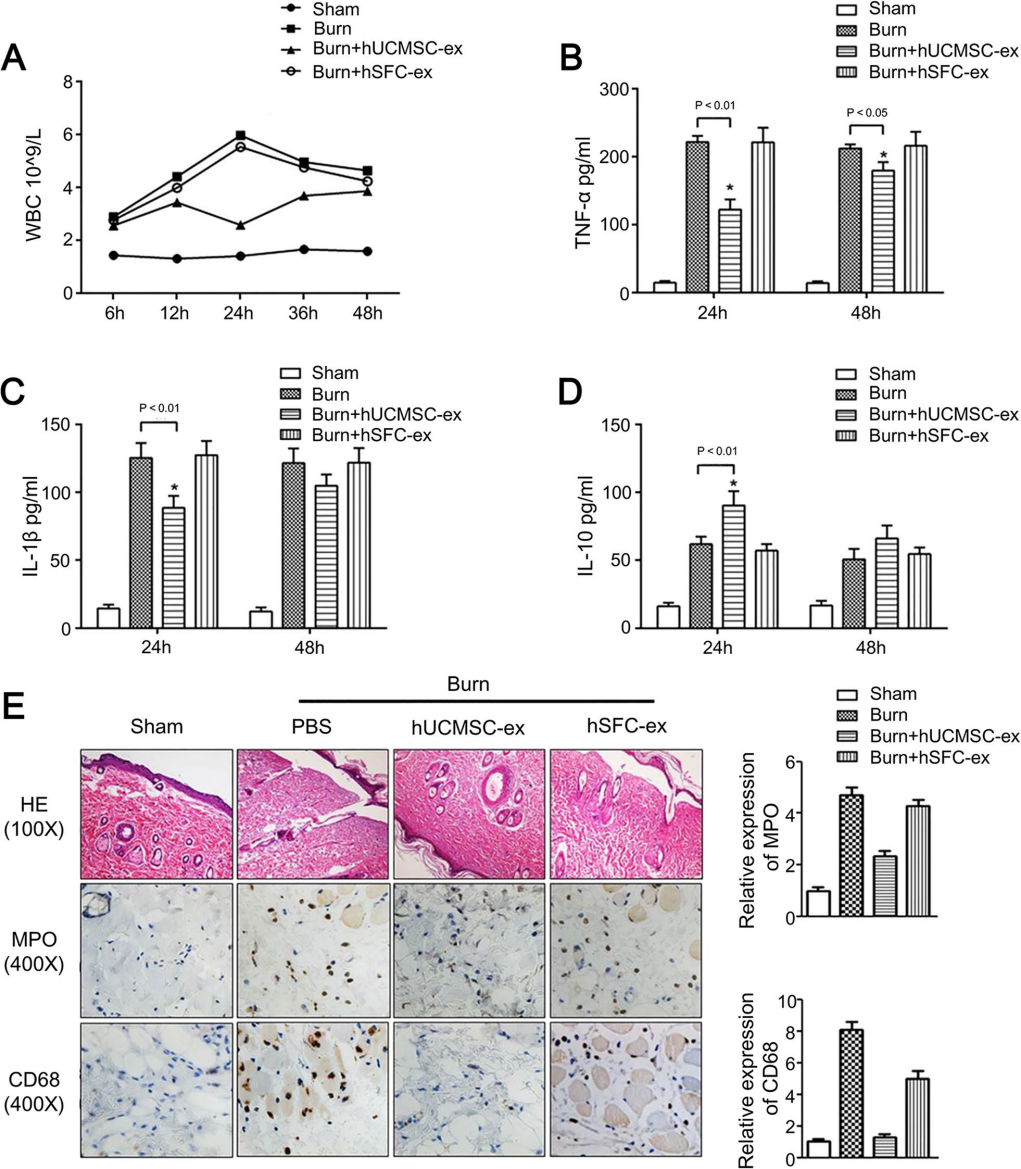
The inflammation in burn rats was alleviated by hUCMSC exosomes (hUCMSC-ex) [45]. **A** The number of WBC in sham and burn rats treated with PBS, hUCMSC-exosomes, or hSFC-exosomes; **B**–**D** the expression levels of TNF-α, IL-1β and IL-10 in different groups; **E** histological images and the positive neutrophils (MPO) and macrophages (CD68) staining in burn wounds. The quantitative assay of MPO and CD68 was shown. hSFC: human skin fibroblast cell; WBC: white blood cells; PBS: phosphate-buffered saline; TNF-α: tumor necrosis factor-α; IL-1β: interleukin-1β; IL-10: interleukin-10. Reprinted from EBioMedicine, Vol (8), Li et al., Exosome Derived From Human Umbilical Cord Mesenchymal Stem Cell Mediates MiR-181c Attenuating Burn-induced Excessive Inflammation, p.72–82, copyright 2016, with permission from Elsevier

Different cargoes in exosomes show therapeutic effects in tissue regeneration, such as cell proliferation, angiogenesis, and inflammation. For example, Choi et al. identified that miRNAs in the exosomes of AD-MSCs could suppress genes associated with cell senescence, thus improving the proliferation and migration of skin fibroblasts [43]. Gangadaran et al. revealed an angiogenic property of EVs containing abundant miRNA-210-3 ps and VEGF proteins [44]. Li et al. evaluated the levels of inflammatory factors (TNF-α, IL-1β and IL-10) and inflammatory cells in the burn rats treated with UC-MSCs-derived exosomes, aiming to investigate the effects of exosomes in cutaneous inflammation (Fig. 8) [45]. The administration of MSCs-derived exosomes could alleviate the inflammation induced by burn injury. The authors further revealed that exosomes overexpressing miRNA-181c were able to suppress Toll-like receptor 4 pathway to regulate inflammation [45]. Thus, miRNA-181c is considered to be a potential target to restrict inflammation and promote wound repair. Therefore, exosomes have positive effects on tissue regeneration, but the functional molecules delivered in exosomes and their action mechanisms need to be studied further.

The cell-free therapy sheds new light on tissue regeneration by replacing MSCs with exosomes, which may overcome poor cell engraftment and reduce the risks of immune rejection in cellar therapy. Additionally, exosomes can be stored safely and easily relative to MSCs. Exosomes retain the functions of their parent cells and can be modified to deliver cargoes to exert therapeutic effects, which holds a promising future in clinical application.

### Delivery methods

Ensuring the survival and function of cells during delivery is also a strategy to increase cell efficacy. Direct local injection and intravenous infusion are common methods to deliver MSCs to the injury site, having shown successful outcomes in wound repair. However, there are some drawbacks due to the influence of delivery routes on cell viability and function. The direct injection could affect the integrity of the cell membrane due to the mechanical stresses caused by the syringe needle. Besides, the connection between cells and the extracellular matrix is interrupted, causing apoptosis. This method also fails to achieve a homogeneous distribution of cells in the injury site. Intravenous infusion is easier to implement and less invasive than the direct local injection. However, cells that reach the target wound site are limited because some cells are entrapped in the lungs during intravenous infusion [46]. Therefore, some novel delivery methods have been developed to reduce cell death and improve transplantation efficiency.

The application of a specific biomaterial scaffold has shown great promise in cell transplantation. The scaffold can increase the delivery efficiency, providing support for cell survival as a physical architecture. It possesses outstanding compatibility and can interact with MSCs favorably, thereby making the cell living environment more suitable. MSCs delivered in scaffold have enhanced retention and proliferation, which are associated with the type of biomaterial. A study compared the effects of four different biomaterials seeded with MSCs in wound healing [47], showing that the cell activity and paracrine function were varied with different scaffolds. Both natural and synthetic biomaterials are used to deliver MSCs as scaffolds, and their combined application exhibits new prospects in skin regeneration. Chu et al. designed a collagen hybrid scaffold composed of polyethylene glycol and graphene oxide, which promoted angiogenesis and collagen deposition in diabetic skin repair [48]. This novel scaffold provided a superior environment for cell attachment, proliferation, and differentiation. Composite scaffolds with different biomaterials need to be more investigated to exert unique material characteristics.

**Fig. 9:**
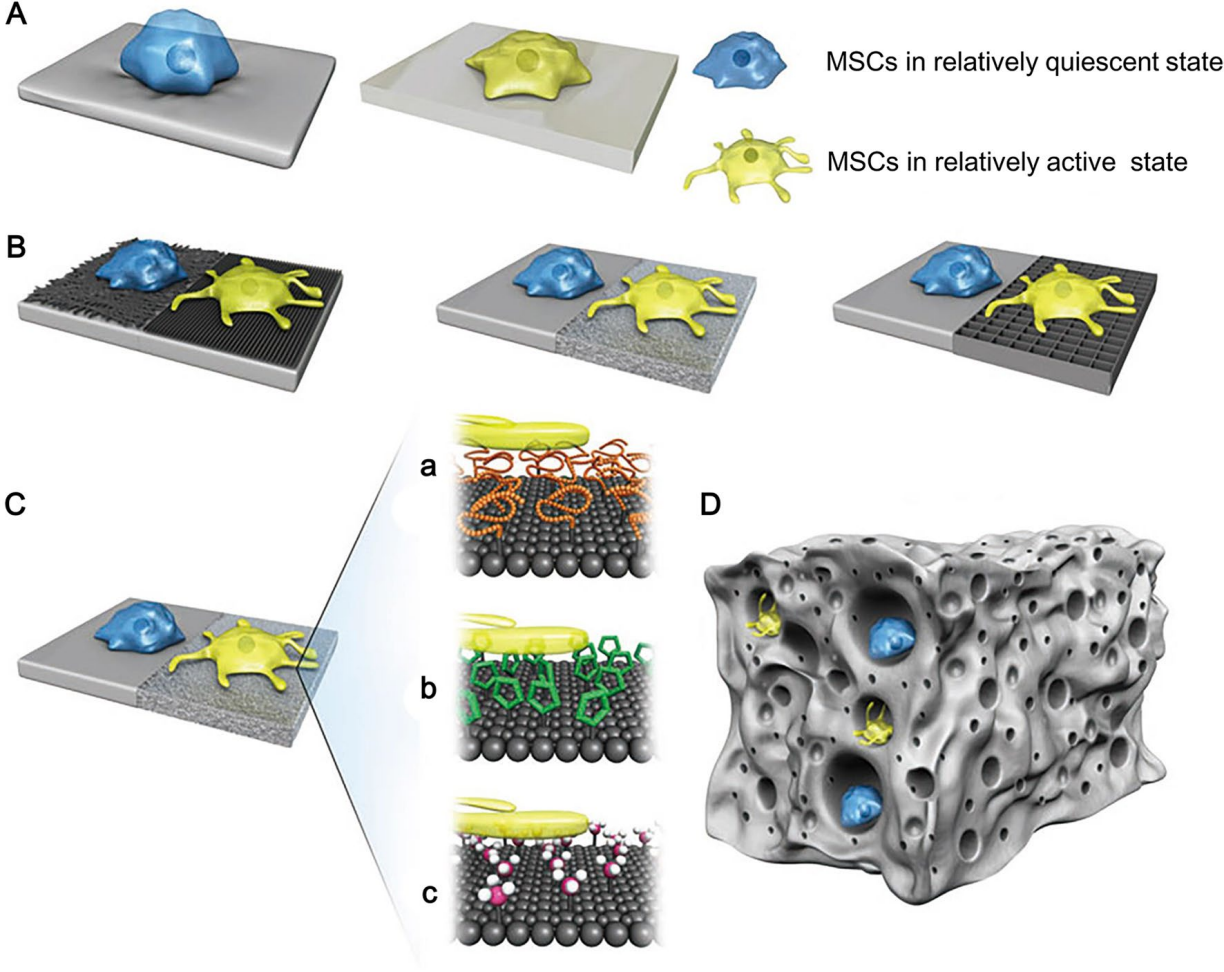
The functions of MSCs are affected by the properties of biomaterials [49]. **A** The effects of biomaterial stiffness on MSCs; **B** the effects of surface topography of biomaterials on MSCs; **C** the effects of surface chemistries of biomaterials on MSCs, such as (a) proteins, (b) pharmaceutical molecules, and (c) functional groups; **D** the effects of pore size on MSCs. Reproduced from the article by authors Chen et al. [49], copyright 2019. The publisher for this copyrighted material is Mary Ann Liebert, Inc., publishers

Different microstructures of biomaterials have different effects on the growth and function of MSCs, such as the pore size, stiffness, topography, and chemistries of biomaterials (Fig. 9) [49]. For example, Bonartsev et al. demonstrated that pore size of polymer scaffolds was a crucial factor affecting cell growth and differentiation [50]. The uniform pore size of scaffolds is beneficial for cell differentiation, while the widely distributed pore size is suitable for cell growth [50]. Additionally, changes in the stiffness and surface characteristics of the scaffolds can result in the different paracrine functions of MSCs. The immunomodulatory protein production of MSCs is increased by regulating the scaffold stiffness [51]. Stiffness is considered as a switch to modulate related signal pathways of immunomodulation [51]. Modulating surface characteristics of the scaffold, such as the fibrous topography, shows more secretion of proangiogenic and anti-inflammatory cytokines in AD-MSCs relative to the raw microplates [52]. The enhancement of cell paracrine secretion accelerated wound healing through the recruitment and polarization of macrophages [52]. Thus, the mechanical properties of the scaffold can be harnessed to promote cell function and delivery efficiency. Besides, incorporating chemotactic factors, functional groups, or side chains with scaffolds through chemical modification is also a practical approach to deliver MSCs and increase cell efficacy. According to the excellent properties of biomaterials, physical or chemical modifications can further improve the efficiency of cell delivery.

According to the application of biomaterial scaffold, an advanced strategy to encapsulate cells in a semisolid membrane has been explored. Cells are in relative isolation from the external environment and maintain normal physiological activity. Encapsulated MSCs in composite microgels exhibited increased cell viability and promoted anti-oxidant functions in oxidative stress conditions [53]. Both the reactive oxygen species (ROS) scavenging ability of microgels and the encapsulation method protected MSCs from the damage of oxidative stress. The immunomodulatory capacity of encapsulated MSCs after treatment of inflammatory cytokine was assessed using a microfluidic device to encapsulate cells in the alginate coating, showing an increased expression of immunomodulatory-associated genes [54]. In addition to modulating the immune response, this encapsulation system also extended cell retention. Hence, the combined application of biomaterial scaffold and cell encapsulation can improve the delivery efficiency of MSCs.

### Preparations of host tissue

**Fig. 10:**
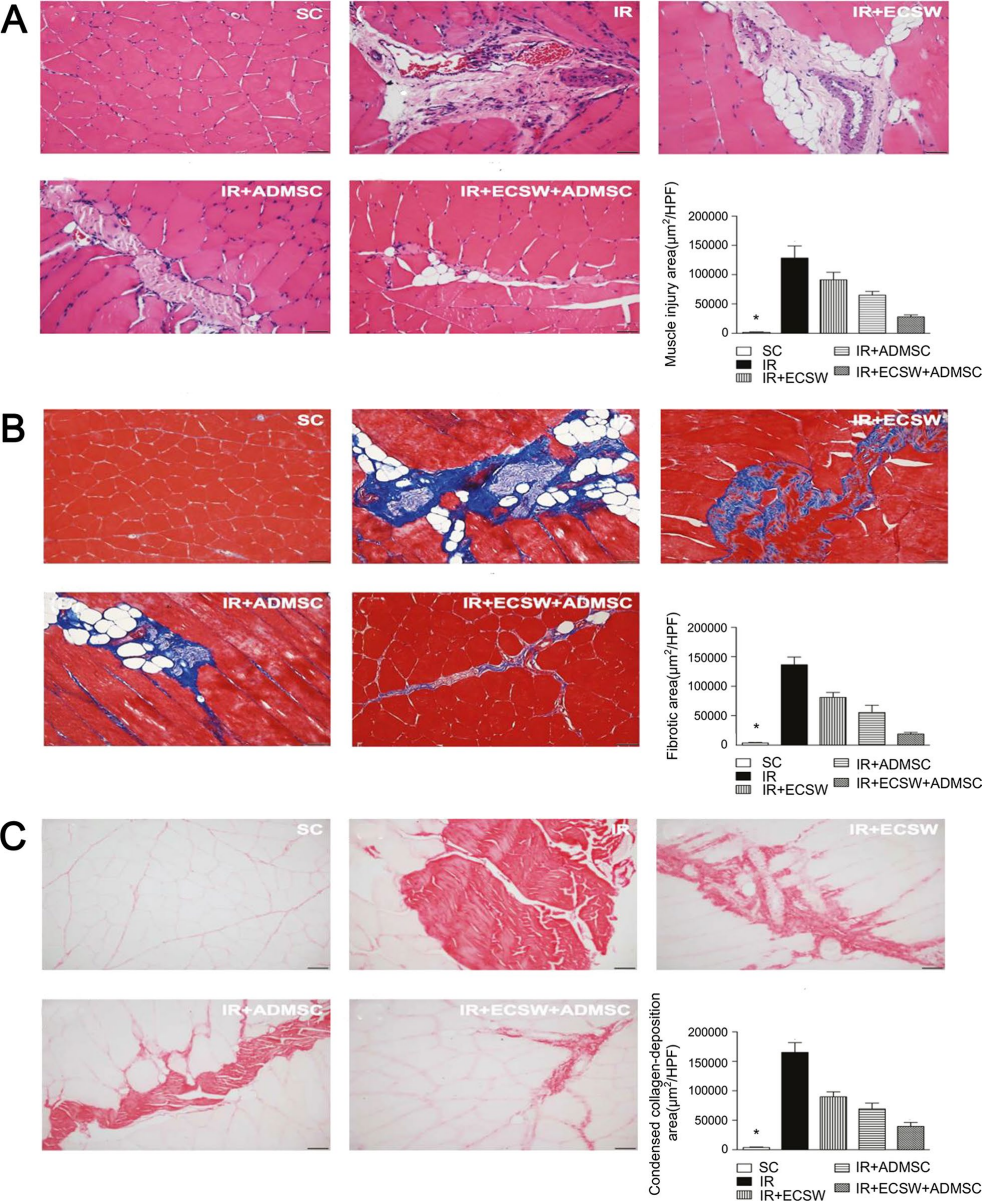
The effects of combined therapy of MSC and ECSW on ischemic muscle injury [55]. The images and quantitative analysis of muscle injury area (**A**), fibrotic area (**B**), and collagen-deposition area (**C**) in different groups. HPF: high-power field; SC: sham control; IR: ischemia–reperfusion; ECSW: extracorporeal shock wave; ADMSC: adipose tissue-derived mesenchymal stem cells. Reproduced from the article by authors Yin et al. [55], copyright 2018, Yin et al.

Apart from the cell precondition, researchers also considered the preparations of host tissue environments to increase the adaptability of cells to harsh environments. Physical methods can be used for host tissue preconditioning. Combined with MSC therapy, extracorporeal shock wave (ECSW) can significantly reduce the muscle damage, fibrosis, and collagen deposition in a rat model of ischemic muscle injury, proving to have therapeutic effects on tissue regeneration (Fig. 10) [55]. Besides, the cellular expressions of inflammatory are decreased, and the expressions of angiogenesis markers are increased, indicating a reduction of inflammation after receiving this combined therapy. Weihs et al. revealed the molecular mechanism by which ECSW exerts its positive effects in wound healing [56]. ECSW facilitated the cell proliferation and healing rate by activating extracellular signal-regulated kinase (ERK) signaling. This study provided a new understanding of the clinical use of ECSW. Furthermore, pharmacologic preconditioning of recipient tissue is also effective in creating a favorable environment for cell growth. A study of myocardial tissue repair reported that vasodilatory drugs had a beneficial effect on cell delivery [57]. However, the effect was not caused by the vasodilatory function of drugs, and the underlying mechanism was not clear. Thus, the role of vasodilatory drugs in wound regeneration and other drugs with different pharmacological effects in promoting wound healing need more exploration.

## Discussion

The therapeutic efficacy of stem cells has been investigated intensively in wound regeneration. Different types of stem cells have their unique characteristics to promote wound healing. Over the past few years, the role of MSCs in wound healing has been identified, and studies about MSCs have made significant strides. This paper is also based on MSCs to discuss improving the efficacy of stem cells in wound regeneration.

Cell characteristics, delivery process, and host factor all influence cell survival and effectiveness. Some strategies are proposed to increase cell efficacy and prevent cell death in tissue regeneration. For the preparation of MSCs, the first thing is selecting the appropriate source of cells according to the needs of the situation to achieve the desired recovery effect. According to the stage of wound healing, priority is given to select cell sources and subpopulations that are beneficial in combating inflammation, stimulating angiogenesis, promoting matrix deposition, or reducing scar formation. Allogeneic or syngeneic MSCs applying to tissue regeneration is determined by the specific circumstance. The immune response induced by syngeneic MSCs is negligible, but their use is limited in emergencies. As for allogeneic cells, the age of the donor and health condition needs to be assessed. Various forms of preconditioning approaches exhibit satisfactory outcomes by enhancing the resistance of MSCs against the hostile environment or reducing the environmental damage to cells. Cell preconditioning and host tissue preconditioning both are effective methods to maintain cell retention and increase cell efficacy. Culture condition with low oxygen and nutrition enables cells more adaptable by mimicking the host tissue environment. The 3D aggregation of MSCs can better preserve cell properties. Co-culturing MSCs with other cells can increase the specific therapeutic properties of MSCs. Modifying the target gene and manipulating related microRNA prolongs cell survival and enhances the paracrine function. Replacing the cells with their secretome represents a new direction for cell-free therapy. In the delivery process, the application of biomaterial scaffolds reduces mechanical pressure and preserves intercellular communication. The encapsulation of MSCs provides protection for maintaining cell biological activity.

These strategies from different respects could improve cell efficacy in wound healing. Although great progress has been made in cell therapy, several issues need to be considered to achieve the clinical application of stem cells: firstly lack of effective biomarkers of stem cells to define specific characteristics from different sources. Heterogeneous populations of stem cells exhibit differences in functions. Identifying effective biomarkers is also helpful in dynamically monitoring cell activity. Secondly, current researches have not determined the optimal type and source of stem cells for tissue regeneration due to differences in experimental design, animal models, operating procedures, and the dose and timing of the stem cells applied. A standardized process for using stem cells needs to be established to facilitate future scientific normative comparisons of different stem cells. Thirdly, the expression and changes of various molecules participating in the physiological process of wound healing remain unclear. The physiological changes in the microenvironment at the wound site can be understood deeper by clarifying the communication between cells and molecules. Finally, although the stem cells possess immunosuppressive properties, stem cells are considered to elicit varying degrees of immune responses in the recipient. More animal model experiments need to be taken to obtain more detailed experimental data in immunology. A controlled immune response can significantly reduce the adverse effects of stem-cell therapy.

Future efforts are required to understand the underlying mechanism of stem cells for the therapeutic effects in wound regeneration. The way cells behave and how they interact with the surrounding environment remain unclear. The roles of paracrine molecules of cells need to be clarified. It is also necessary to determine the host microenvironment and the effects of this microenvironment on the cell. Controlling the microenvironment to be favorable to the cell by the pretreatment of the host tissue is an effective approach. Besides, exploiting more suitable delivery materials or developing more efficient delivery methods is desirable to maintain cell survival and enhance cell function. Preparation of the cell, host tissue, and delivery process should be designed more carefully to unleash a higher cell therapeutic potential. Cell transplantation and survival conditions will be enhanced by different strategies, thus contributing to efficient stem cell-based therapy. Moreover, non-cell therapy with the application of cell secretomes is another new direction.

## Conclusion

Cutaneous wound regeneration has been a topic of great concern in recent years. Various traditional and emerging methods are applied to enhance wound repair, in which stem cell therapy has attracted much attention. The main wound healing process has been described, while the underlying mechanism by which stem cells act on wound healing has not been completely elucidated. The therapeutic effects of stem cells are limited by poor viability and low delivery efficiency. Therefore, diverse strategies are proposed to maintain cell retention and improve cell function. Before stem cells are transplanted to the recipient, both cell and recipient can be prepared to achieve a higher therapeutic outcome. The selection of cell type and source, the identification of cell subpopulation and donor, and the investigation of different preconditioning treatments and genetic modification approaches can guarantee enhanced cell efficacy. The biocompatible materials scaffold can increase cell delivery efficiency. Maintaining the harmony between the recipient and transplanted cell is an important goal. More detailed research on the exosomes of stem cells will open up a possibility of cell-free therapy, providing an optimistic future in wound regeneration.

## Data Availability

All data generated or analyzed during this study are included in this published article.

## Abbreviations

ESCs: Embryonic stem cells
ASCs: Adult stem cells
iPSCs: Induced pluripotent stem cells
MSCs: Mesenchymal stem cells
ECM: Extracellular matrix
ISCT: International Society for Cellular Therapy
BM: Bone marrow
AD: Adipose tissue
WJ: Wharton’s jelly
UC: Umbilical cord
AF: Amniotic fluid
PL: Placenta
3D: Three-dimensional
AM: Amniotic membrane
CV: Chorionic villi
PBS: Phosphate-buffered saline
VCAM-1: Vascular cell adhesion molecule 1
TGF-β: Transforming growth factor-β
TNF-α: Tumor necrosis factor-α
IL-1β: Interleukin-1β
IFN-γ: Interferon-γ
PRP: Platelet-rich plasma
FGF-2: Fibroblast growth factor-2
IGF-1: Insulin-like growth factor 1
TGF-β1: Transforming growth factor-β1
VEGF: Vascular endothelial growth factor
SDF-1: Stromal-derived factor-1
CXCR4: C-X-C chemokine receptor 4
Ang1: Angiopoietin-1
miRNA: MicroRNA
MVB: Multivesicular body
EV: Extracellular vesicle
ROS: Reactive oxygen species
ECSW: Extracorporeal shock wave
ERK: Extracellular signal-regulated kinase
